# Equine-assisted therapy effectiveness in improving emotion regulation, self-efficacy, and perceived self-esteem of patients suffering from substance use disorders

**DOI:** 10.1186/s12906-023-04191-6

**Published:** 2023-10-13

**Authors:** Nagwa Souilm

**Affiliations:** Faculty of Nursing, Beni-Suef University, Beni-Suef, Egypt

**Keywords:** Equine-assisted therapy, Emotion regulation, Self-efficacy, Self-esteem, Substance use disorders (SUD)

## Abstract

Substance Use Disorders (SUD) is a universal overwhelming public health problem and is associated with other psychological and mental health ailments such as emotion regulation, perceived self-esteem, and self-efficacy problems. Complementary and alternative medicine may be beneficial. The aim of this study was to assess the effectiveness of equine-assisted therapy in improving emotion regulation, self-efficacy, and perceived self-esteem among patients suffering from substance use disorders. It was carried out using a randomized controlled trial design at Behman hospital, Cairo, Egypt. It included 100 patients suffering from SUD attending the setting, equally randomized into an intervention group to receive the equine assisted therapy and a control group to receive the regular care. Data were collected using a self-administered questionnaire with standardized tools for assessment of emotion regulation, General Self-Efficacy (GSE), and perceived self-esteem. The intervention group received weekly equine-assisted therapy sessions over 6 weeks in addition to their standard regular therapy. Patients in both groups had similar demographic and SUD characteristics, as well as baseline scores of reappraisals, suppression, GSE and perceived self-esteem. At post-intervention, the intervention group had significant improvements in all these scores in comparison with the control group, as well as their baseline. The multivariate analysis identified the study intervention as a significant positive predictor of the reappraisal and GSE scores, and a negative predictor of the suppression and perceived self-esteem negative score. In conclusion, equine assisted-therapy as a complementary treatment in patients suffering from SUD is effective in improving their emotion regulation, self-efficacy, and perceived self-esteem. A wider use of this approach is recommended in SUD patients along with provision of needed facilities and resources, and training nurses in its administration. Further research is proposed to assess its long-term effectiveness. The clinical trial was registered in the “Clinical Trials.gov Protocol Registration and Results System (PRS);” registration number is (05632185/2022) and the full date of first registration is 10/11/2022.

## Introduction

Substance Use Disorders (SUD) is a term replacing the diagnoses of abuse and dependence adopted in DSM-4 and DSM-5 [1]. It is an overwhelming public health problem throughout the world, with increasing trends throughout the last few decades [2]. This could be attributed to many social determinants such as feeling of insecurity, trauma, severe distress, and repression [3]. It involves intricate interactions between the life experiences of the affected person and his/her brain paths and circuits, and the genetic and environmental factors [4]. The disorder is associated with high rates of mortality and morbidity even more than any other preventable conditions [5].

The patients suffering from SUD are often affected by other psychological and mental health problems. For instance, many of these patients may have their ability to regulate emotions disrupted, and this could be the cause underlying their SUD. They are unable to adequately adjust positive and painful emotions in response to difficult situations, or to regulate their emotions. Therefore, they may try substance use to help themselves reach a balance of emotions [6].

Moreover, SUD patients may have a feeling of low perceived self-esteem [7], so that improving their self-esteem proved to be of great help in the success of their treatment [8]. Similarly, self-efficacy plays an important role in the proper management of SUD since it reflects a person’s ability to use own skills in a certain situation to achieve a certain goal, and thus it was shown to improve the prognoses of SUD patients [9].

Although SUD is considered a stigma in many cultures [10], it is a disease that needs to be treated with no discrimination against affected patients [11]. Despite the high prevalence of SUD worldwide, both in developed and developing countries, a majority of patients suffering from this disorder do not receive proper treatment [12]. Treatment modalities are numerous, and they tend to modify patients’ behaviors ultimately leading to abstinence [13]. Meanwhile, recent cognitive-behavioral treatment modalities try to foster patients’ coping with stressful events and various tempting-craving cues. Examples are reconsolidation-based and mindfulness-based interventions, virtual-reality, and pharmacologic augmentation approaches [14]. Complementary and alternative medicine seem to gain more attention in the management of SUD [15]. Failure of treatment and relapse is common in SUD. However, a systematic review concluded that there is no universal consensus upon what constitutes a relapse, and how to distinguish early from late relapse [16].

Equine-Assisted Therapy (EAT) is a treatment approach where patients do interact with horses with or without actual mounting activities or riding [17]. The approach is based on helping the occurrence of a regulated purposeful interaction between a physically able person and a live horse through a facilitator, often a mental health professional certified in animal-assisted therapy. It is based on developing a successful communication between the patient and the horse [18]. It helps through having a common focus, improving motivations and self-confidence, fostering positive emotions, in addition to the benefits of physical activity [19]..

Furthermore, research proposed that such interaction with the horses in equine assisted therapy involves trust, patience, and respect. It could alleviate patients’ anxiety and improve control of emotions, with less tendencies to disruptive behavior. It was also shown to improve balance, trust, wellbeing, self-esteem, and self-efficacy among patients suffering a wide spectrum of psychological and mental disorder [20, 21].

### Significance of the study

Substance use disorders represent a major public health problem worldwide as well as in Egypt. The prevalence rates have increasing trends. Treatment modalities are diverse and have a wide range of success rates. Although novel approaches as equine-assisted therapy show encouraging results, there is still deficient research addressing it, particularly in Egypt.

### Aim of the study

To assess the effectiveness of equine-assisted therapy in improving emotion regulation, self-efficacy, and perceived self-esteem among patients suffering from substance use disorders.

### Research hypotheses

Compared with control group patients, (1) Patients suffering from substance use disorder (SUD exposed to equine-assisted therapy will have significantly better emotion regulation (higher reappraisal and lower suppression scores); (2) Patients suffering from substance use disorder exposed to equine-assisted therapy will have significantly higher GSE scores; (3) Patients suffering from substance use disorder exposed to equine-assisted therapy will have significantly better perceived self-esteem (lower scores)..

## Participants and methods

### Research design

The design used in carrying out the study was randomized controlled trial. Since the intervention (exposure to and dealing with horses vs. not) could not be hidden from participants or investigators given its nature, the trial was open-label, i.e. not double blind.

### Setting

The study was conducted at Behman hospital, Helwan, Cairo, Egypt. It was inaugurated in 1940 as the first Egyptian private psychiatric hospital. With a 210-bed capacity, the hospital is considered as one of the biggest medical centers in Cairo, Egypt. The hospital provides care for all sectors of patients in Egypt, especially in Cairo. It uses multidisciplinary approaches to treatment plans, with complementary therapies, and provision different intervention options designed to enhance patients’ therapeutic journeys. Available alternative treatments include art therapy, dance movement and music therapy, yoga and physiotherapy, and equine psychotherapy. The hospital is the pioneer of equine psychotherapy in Egypt.

### Participants

**Fig. 1:**
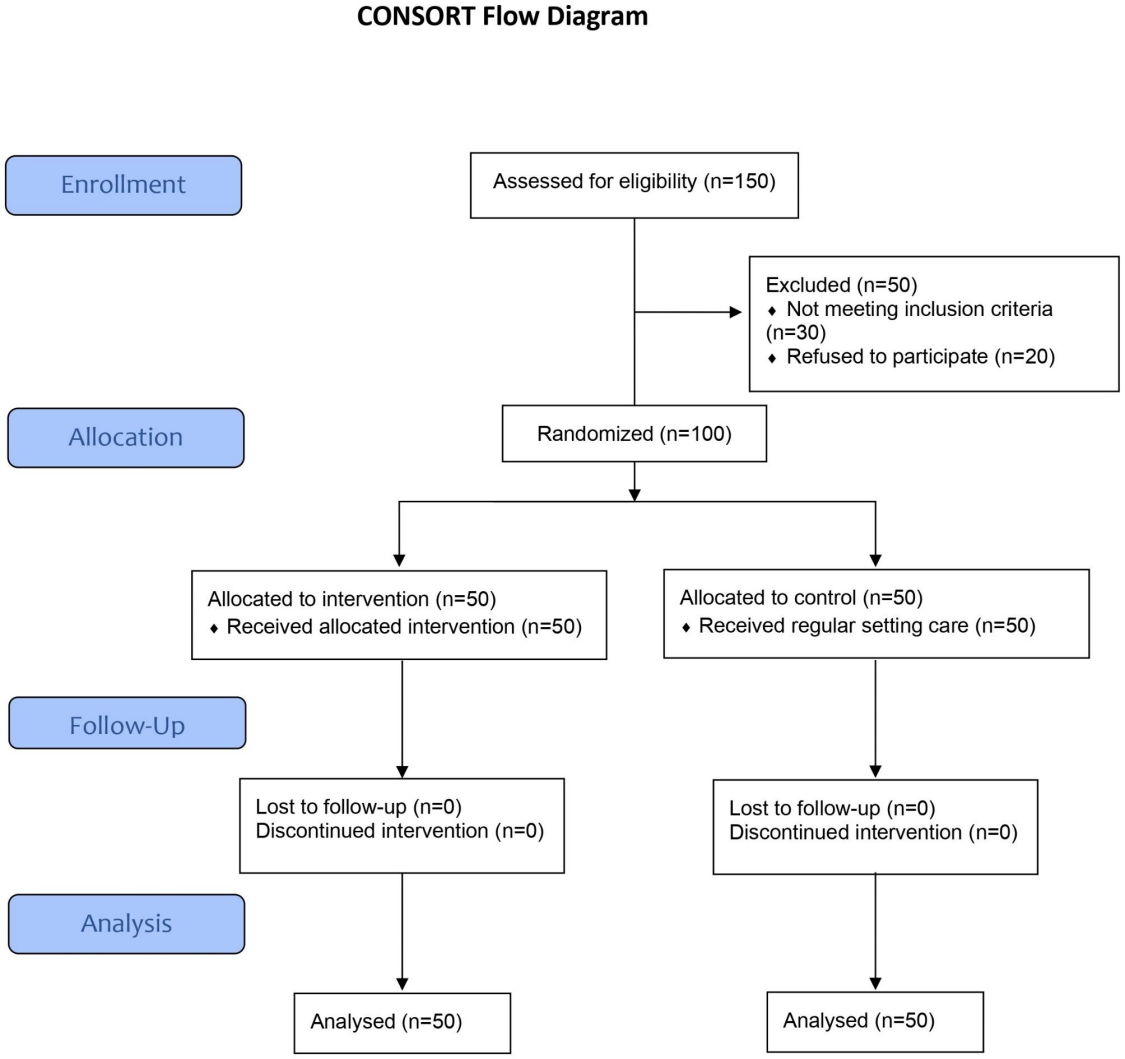
The flowchart of the study

The study population consisted of patients suffering from substance use disorder receiving their care at Behman Hospital during the time of the study. The inclusion criteria were being adult diagnosed with substance use disorder, hospitalized for at least one month in the study hospital, and physically able to deal with horses. Those patients suffering from any other physical or psychological ailments preventing them from active participation in the intervention were excluded. Using the G*Power sample size software program, the required sample size per group was 50 to detect scores’ improvement with a medium effect size (0.60) at 95% level of confidence and 80% power accounting for an expected dropout rate of about 20%. A total of 150 patients were enrolled, of which 30 were excluded for not meeting the eligibility criteria, and 20 for refusing participation (Fig. 1). The remaining 100 were equally randomized, using simple random numbers computer program, into an intervention group to receive the equine assisted therapy and a control group to receive the regular care used in the setting. A list of the recruited 100 patients fulfilling the eligibility criteria was prepared with enumeration from 1 to 100. Computer-generated random numbers were obtained for the two groups. Each patient was assigned to the corresponding group (intervention or control) according to his/her number in the list..

### Data collection tools

Data were collected using a self-administered questionnaire with standardized tools for assessment of emotion regulation, General Self-Efficacy, and perceived self-esteem, in addition to a section for the demographic and disease characteristics developed by the researchers. This covered data about patient age, gender, university education, marital status, residence, job status, income, as well as age at start and duration of substance of use, length of hospital stay, number of substances used and their types. The questionnaire was reviewed by five experts in psychiatric nursing for the completeness of the demographic and medical parts and for the relevance of the three standardized scales.

The first tool was the emotion regulation questionnaire developed by Gross and John. [22] It was used to assess how respondents tend to regulate their emotions positively through cognitive reappraisal, or negatively through expressive suppression. It consists of ten statements: 6 for cognitive reappraisal (such as “*When I want to feel more positive emotion, I change the way I’m thinking about the situation*”), and 4 for expressive suppression (4 statements such as “*When I am feeling negative emotions, I make sure not to express them*”). The responses are on a 7-point Likert-type scale ranging from “strongly disagree” to “strongly agree.” The scores of each dimension are summed giving a total score ranging from 6 to 42 for the appraisal dimension and from 4 to 28 for the suppression dimension. A higher score indicates more appraisal or more suppression. Scoring is kept continuous according to tool instructions. The tool demonstrated high validity and reliability in previous studies [23, 24]. Additionally, the reliability of this scale was assessed through its internal consistency, and it proved to be high with Cronbach’s alpha coefficients 0.74.

The second tool was the GSE scale developed by Schwarzer and Jerusalem [25]. It also has 10 statements on a 4-point Likert type scale ranging from “not at all true” (scored 1) to “exactly true” (scored 4). Examples of statements are: “*It is easy for me to stick to my aims and accomplish my goals*,” and “*I can solve most problems if I invest the necessary effort*.” A total score ranging from 10 to 40 is obtained simple summation of items’ scores. A higher score means more GSE. The reliability of the tool as well as its concurrent and predictive validity were documented [26]. Its reliability in the present study was high with Cronbach’s alpha coefficient 0.96.

The third tool was the self-esteem test developed by Sorensen [27]. The tool has 50 statements such as: “*I often feel like I don’t know what is expected of me*,” and “*I am not very aware of my feelings*.” The respondent is asked to check the statements he/she feels true for him/her. The number of items checked is calculated, ranging from zero to 50. A higher number indicates lower self-esteem. The tool was validated and showed good reliability [28]. The reliability of this scale was high with Cronbach’s alpha coefficients 0.78.

### Administrative and ethical considerations

The researchers got the required official permissions to carry out study through a letter addressed from the Dean of the Faculty of Nursing, Beni-Suef University to the hospital directors explaining the study aim and procedures, with a copy of the self-administered questionnaire. The study protocol was approved by the Ethical Committee in faculty of medicine, Beni- Suef university, Egypt; approval number (0006240). All Helsinki Declaration research ethics principles were complied with. An informed written consent was obtained from each patient at recruitment. The clinical trial was registered in the “Clinical Trials.gov Protocol Registration and Results System (PRS);” registration number is (NCT05632185/2022) and the full date of first registration is 30/11/2022. Available at:https://ichgcp.net/clinical-trials-registry/NCT05632185.

The researchers clearly explained the aim of the study and its maneuvers to every patient, along with information about their rights before inviting them to participate. They were clearly informed that participation is totally voluntary, and that they may withdraw any time with no reason required. They were also reassured about the confidentiality of any obtained information. An informed written consent was obtained from each patient at recruitment.

### Pilot study

A pilot study was carried out on a sample of ten patients representing 10% of the main study sample size in order to assess the clarity of the tools and the practicability of the study procedures. The tool was fine-tuned according to the results of the pilot. These ten patients were not included in the main study to avoid any contamination bias.

### Fieldwork

Upon obtaining official permission, the researchers started the patients’ recruitment process according to the eligibility criteria. A total of 150 patients were screened, and of these 100 were enrolled. They were asked to fill in the data collection forms. The filled forms were collected by the researchers and reviewed for completeness. The patients were then randomized into the equine-assisted therapy intervention group and the control group.

The intervention group patients received weekly equine-assisted therapy 40–60 min sessions over a period of 6 weeks in addition to their standard regular therapy. In the frst session, the researchers gave a presentation of the program’s aim and objectives, its sessions, as well as the procedures to be followed throughout the intervention. The second to the sixth sessions were practical sessions. An equine therapy session typically consists of specific tasks and challenges to overcome, with the aid and guidance of horses. There is no riding involved and no specific skills are necessary to take part in these sessions, e.g., the session might involve the patient successfully leading the horse from one spot to another, or putting a halter on the horse, grooming, or feeding. By the end of the session, the patient discusses with the therapist what ideas and thoughts he/she used to complete the task. Once the session ends, the patients along with their therapists reflect on the experience and the emotions that were evoked by the interaction.

The control group had the same standard regular treatment modalities with the only exception of not having the equine-assisted therapy. At the end of the intervention, all patients in both groups were asked to fill in the self-administered questionnaire in order to assess the effectiveness of the equine-assisted therapy. Thus, data were collected at two points in time. To compensate for the potential bias of the open-label design where the intervention (due to its nature) could not be concealed from the patients and the researchers, blinding was applied during the processes of data collection and analysis. Therefore, the data collectors who dealt with the patients and the data analyst did not know whether the information came from a patient in the intervention or in the control group. The fieldwork lasted from February to July 2022..

### Statistical analysis

Categorical variables were described as frequencies and percentages, while numeric ones were presented as means, standard deviations, and medians. The reliability of the scales used was examined using Cronbach alpha coefficients. Chi-squared tests were applied in categorical variables’ analyses. Student t-test and paired t-test were used for comparisons of numeric variables (scores) between and within groups after checking for required assumptions. Multiple linear regression analyses were carried out to identify the independent predictors of the scores of reappraisal, suppression, GSE, and self-esteem. Statistical significance was considered at p < 0.05. Data entry and analyses were conducted using IBM SPSS Statistics for Windows, version 20 (IBM Corp., Armonk, N.Y., USA).

## Results

Table 1 demonstrates that patients in the study and control groups have similar demographic charateristics. The highest percentages were in the age group 30-<50 years, with equal gender distribution in the study group and slightly more men in the control group (56.0%). The intervention group had slightly less university graduates (p = 0.03).

**Table 1:** Demographic characteristics of patients in the intervention and control groups

	Groups				X^2^ test	p-value
	Intervention (n = 50)		Control (n = 50)			
	No.	%	No.	%		
Age:						
< 30	18	36.0	16	32.0		
30-	25	50.0	29	58.0	0.75	0.69
50+	7	14.0	5	10.0		
Gender:						
Male	25	50.0	28	56.0		
Female	25	50.0	22	44.0	0.36	0.55
University degree:						
No	24	48.0	14	28.0		
Yes	26	52.0	36	72.0	4.24	0.03*
Marital status:						
Unmarried	27	54.0	22	44.0		
Married	23	46.0	28	56.0	1.00	0.32
Residence:						
Urban	38	76.0	40	80.0		
Rural	12	24.0	10	20.0	0.23	0.63
Job status:						
Unemployed	25	50.0	16	32.0		
Working	25	50.0	34	68.0	3.35	0.07

(*) Statistically significant at p < 0.05

Table 2 shows that in more than a half of the patients in both groups the age at start was 15 years and older. The duration of substance use was slightly longer (5 years or more) in the control group (52.0%) compared to the study group (46.0%). The majority of the patients in the two groups were using 2 or more substances, most commonly opiates/narcotics, and sedatives/ tranquillizers. No statistically significant differences were revealed between the two groups.

**Table 2:** Characteristics of substance use among patients in the intervention and control groups

	Groups				X^2^ test	p-value
	Intervention (n = 50)		Control (n = 50)			
	No.	%	No.	%		
Age at start of drug use (years):						
< 15	22	44.0	20	40.0		
15+	28	56.0	30	60.0	0.16	0.69
Duration of illness (years):						
< 5	27	54.0	24	48.0		
5+	23	46.0	26	52.0	0.36	0.55
Length of hospital stay in month (LOS):						
1–2	32	64.0	32	64.0		
3+	18	36.0	18	36.0	0.00	1.00
No. of substances used:						
1	11	22.0	5	10.0		
2+	39	78.0	45	90.0	2.68	0.10
Range	1–4		1–4			
Mean ± SD	2.2 ± 0.9		2.3 ± 0.7		U = 0.20	0.65
Median	2.0		2.0			
Substances used:^@^						
Alcohol	12	24.0	11	22.0	0.026	0.81
Opiates/narcotics	28	56.0	32	64.0	0.67	0.41
Sedatives/tranquillizers	28	56.0	29	58.0	0.04	0.84
Stimulants	26	52.0	24	48.0	0.16	0.69
Hallucinogens	16	32.0	18	36.0	0.18	0.67

*(@) Not mutually exclusive*

As illustrated in Table 3, no statistically significant differences could be demonstrated between the intervention and control groups’ pre-intervention scores of reappraisal, suppression, GSE and perceived self-esteem (negative). At post-intervention, the intervention group had significantly higher scores of reappraisal and GSE and lower scores of suppression and perceived self-esteem (negative). The table also indicates statistically significant increases in the intervention group post-intervention scores of reappraisal and GSE and decreases in their suppression and perceived self-esteem (negative) scores. In the control group, only a statistically significant increase in post-intervention scores of GSE and decrease in the perceived self-esteem (negative) scores were revealed.

**Table 3:** Pre-post-intervention between and within intervention and control groups scores of emotion regulation, GSE, and self-esteem

	Intervention (n = 50)		Control (n = 50)		Student t-test	p-value
	Mean ± SD	Median	Mean ± SD	Median		
Pre-intervention:						
Reappraisal (max 42)	23.4 ± 6.6^a^	24.00	24.2 ± 6.9	25.50	-0.622	0.536
Suppression (max 28)	16.3 ± 5.2 ^b^	17.50	15.6 ± 4.6	16.00	0.676	0.501
GSE (max 40)	20.9 ± 7.8^c^	19.00	21.3 ± 7.2^e^	19.50	-0.240	0.811
Self-esteem^@^ (max 50)	18.0 ± 6.7^d^	18.00	19.0 ± 6.3^f^	18.50	-0.817	0.416
Post-intervention:						
Reappraisal (max 42)	29.1 ± 8.1^a^	33.00	24.0 ± 8.8	27.50	3.022	0.003*
Suppression (max 28)	8.5 ± 6.4^b^	5.00	16.1 ± 5.7	18.00	-6.334	< 0.001*
GSE (max 40)	33.1 ± 7.5^c^	35.00	24.4 ± 7.3^e^	25.00	5.902	< 0.001*
Self-esteem^@^ (max 50)	9.9 ± 5.3 ^d^	7.00	15.7 ± 3.8^f^	18.00	-6.328	< 0.001*

(*) Statistically significant at p < 0.05 (@) negative: higher score indicates lower self-esteem

*Pre-post differences within groups: (a-a, b-b, c-c, d-d, e-e, f-f) Statistically significant at p < 0.05*

The multivariate analysis (Table 4) identified the time (pre-post) as well as the study intervention as the only statistically significant independent positive predictors of the reappraisal score. However, they explain only 5% of the variance of this score. As for the suppression score, the table indicates that the time (pre-post) and the study intervention were its statistically significant independent negative predictors, in addition to the duration of use. The model explains 18% of the variance of the suppression score.

**Table 4:** Best fitting multiple linear regression model for the reappraisal and suppression scores

	Unstandardized Coefficients		Standardized Coefficients	t-test	p-value	95% Confidence Interval for B	
	B	Std. Error				Lower	Upper
Reappraisal score							
Constant	19.95	1.83		10.922	< 0.001	16.35	23.55
Time	2.76	1.10	0.17	2.506	0.013	0.59	4.93
Intervention	2.14	1.10	0.14	1.943	0.053	-0.03	4.31
r-square = 0.05Model ANOVA: F = 5.03, p = 0.007 Variables entered and excluded: age, gender, education, job, marital status, residence, age at start, duration of use, number of substances, LOS							
Suppression score							
Constant	23.59	1.67		14.114	< 0.001	20.29	26.89
Time	-3.70	0.82	-0.29	-4.518	< 0.001	-5.31	-2.09
Intervention	-3.58	0.82	-0.28	-4.371	< 0.001	-5.20	-1.97
Duration of use	-0.87	0.39	-0.14	-2.238	0.026	-1.64	-0.10
r-square = 0.18Model ANOVA: F = 14.50, p < 0.001 Variables entered and excluded: age, gender, education, job, marital status, residence, age at start, number of substances, LOS							

As regards GSE score, Table 5 shows that the time (pre-post) and the study intervention were its statistically significant independent positive predictors, in addition to the number of substances used, although of borderline significance (p = 0.076). Together, they explain 26% of the variance of this score. The table also demonstrates that the time (pre-post) and the study intervention were statistically significant independent negative predictors of the self-esteem negative score. The model explains 26% of the variance of this score.

**Table 5:** Best fitting multiple linear regression model for the GSE and self-esteem scores

	Unstandardized Coefficients		Standardized Coefficients	t-test	p-value	95% Confidence Interval for B	
	B	Std. Error				Lower	Upper
GSE score							
Constant	8.51	2.41		3.536	0.001	3.76	13.26
Time	7.68	1.09	0.43	7.023	< 0.001	5.52	9.84
Intervention	4.28	1.10	0.24	3.908	< 0.001	2.12	6.44
No. of substances	1.24	0.69	0.11	1.783	0.076	-0.13	2.61
r-square = 0.26Model ANOVA: F = 22.37, p < 0.001 Variables entered and excluded: age, gender, education, job, marital status, residence, age at start, duration of use, LOS							
Self-esteem score							
Constant	25.93	1.35		19.243	< 0.001	23.27	28.58
Time	-5.71	0.81	-0.43	-7.029	< 0.001	-7.31	-4.11
Intervention	-3.45	0.81	-0.26	-4.247	< 0.001	-5.05	-1.85
r-square = 0.26Model ANOVA: F = 33.72, p < 0.001 Variables entered and excluded: age, gender, education, job, marital status, residence, age at start, duration of use, number of substances, LOS							

## Discussion

The present hypothesized that in comparison with control group patients, the patients suffering from substance use disorder exposed to equine-assisted therapy will have significantly better emotion regulation (higher reappraisal and lower suppression scores, higher GSE scores and better perceived self-esteem (lower scores). The results demonstrated that the applied study intervention was a significant positive predictor of the reappraisal score and GSE scores and negative predictor of the suppression of the self-esteem negative score. The findings thus lead to acceptance of the set research hypotheses.

The patients were randomized to the intervention and control groups and the randomization process was succesful providing two groups with almost similar demographic and medical charateristics. This is quite important in order to carry out a fair comparison between the two groups. The only difference of statistical significance between the two groups was related to their educational level, where less patients in intervention group were having university education. However, this variable had no confounding effect as shown in the results of the multivariate analyses.

According to the current study findings, the age at start of substance use was mostly 15 years or older among the patients in both groups. This is the age of middle adolescence when the youth tend to experiment with new things and have more risky behaviors. In line with this, a meta-analysis reported that the peak age at start of SUD was 19.5 years [29]. The early onset of substance use has been demonstrated to have an impact on users’ personality and the trajectory of use in a study in Turkey [30]. Moreover, a great majority of these were poly-substance users. This is expected at this age where adolescents like to try different substances to compare and contrast. In line with this, a study in the United States described the trajectories of substance abuse among adolescents and found that the highest percentages were starting in middle adolescence and were using many substances at the same time [31].

As regards the types of substances used, opiates and narcotics as well as sedatives and tranquillizers were most commonly reported among patients in the present study. The findings are in partial agreement with Wakeman et al. [32] whose study in the United States revealed that opioids were the most common type of substances used, followed by alcohol and stimulants. The differences could be attributed to different cultural factors as well as the availability of substances. As for the duration of substance use, around half of the patients in the current study reported 5 years or longer. This would have a significant influence on treatment and its outcomes as demonstrated in another study in the United States [33].

The current study assessed and compared patients’ emotion control (reappraisal and suppression), GSE and perceived self-esteem in the study and control groups before the intervention. The results revealed no significant differences between the two groups, with both having low scores of reappraisals and GSE and high suppression scores. The findings are of great importance given the essential role of emotion regulation in mental health as clarified in a study in Pakistan [6]. Moreover, GSE plays a positive role in the change of the behavior of the patients suffering from substance use disorder, and consequently on the success of their treatment as reported in a study in New Mexico [34]. These authors, through their review of a number of studies, highlighted that self-efficacy was among the major supportive mechanisms for SUD patients enhancing their recovery.

Meanwhile, the present study intervention and control patients’ pre-intervention self-esteem median scores were indicative of slight low self-esteem in at least one half of the samples. This is of major importance in the prognosis of these patients since good self-esteem has been shown to protect against psychological distress among patients under treatment of substance use disorder in a study in Norway [7]. In their study, these authors used the same tool (Rosenberg Self-Esteem Scale) for assessment of self-esteem as in our study, which would make the comparison more valid. They clarified that improving self-esteem would act through the relief of psychological distress of SUD patients. This “salutogenic” effect can help their full recovery.

The implementation of the current study equine-assisted therapy led to statistically significant improvements in the intervention group patients’ emotion regulation, both reappraisal and suppression dimensions, as well as in GSE and perceived self-esteem. The effect of this intervention was confirmed in the results of the multivariate analyses. The effect of the intervention was additive to the effect of the regular treatment modalities, particularly regarding the improvement of GSE and self-esteem as shown in bivariate analyses of the control group patients. In agreement with the benefits of equine-assisted therapy in substance use disorder, Marchand et al. [35] in a study of complementary therapies used in SUD patients highlighted its effectiveness through improving their mindfulness. They used an intervention combining mindfulness training with exposure to nature through recreational sailing.

Furthermore, the results of the current study are in congruence with those reported by Fridén et al. [19] in a study in Sweden. In their qualitative study, the respondents indicated that dealing and interacting with the horses can improve the health capabilities of psychotic patients, enhance their self-confidence and motivation, cultivates positive emotions, and fosters physical activity as well. A similar success of equine-assisted therapy was reported by Aviv et al. [36] where improvement in self-esteem was demonstrated following equine-assisted therapy. However, their study was on ADHD children where this therapeutic approach improved their self-esteem and consequently their executive functions. This adds to the value of equine therapy in the management of a wide spectrum of psychological and mental health problems.

Our study has points of strength and limitations. To our knowledge, this is the first study in Egypt utilizing the equine therapy in the management of SUD patients. The main study limitation is the paucity of similar studies published in peer-reviewed journals, which did not help the researchers to introduce the topic and to discuss the study findings. The bias associated with open-label design could not be avoided, but it was dealt with through blinding the processes of data collection and analysis, which were done by individuals other than the researchers.

## Conclusion and recommendations

The use of equine assisted-therapy as a complementary treatment in patients suffering from substance use disorders is effective in improving their emotion regulation, self-efficacy, and self-esteem.

The study recommends the adoption of this complementary approach in the treatment of SUD patients. The required facilities and resources should be made available in specialized governmental and private SUD treatment centers. The nurses in these facilities need to be trained in its administration. Further research is proposed to assess its long-term effectiveness in SUD patients.

The datasets used and/or analyzed during the current study are available from the corresponding author on reasonable request.

## Data Availability

The datasets used and/or analyzed during the current study are available from the corresponding author on reasonable request.

## Declarations

### Guidelines

All methods were carried out by relevant guidelines and regulations.

## References

[1] Livne O, Shmulewitz D, Stohl M, Mannes Z, Aharonovich E, Hasin D (2021). Agreement between DSM-5 and DSM-IV measures of substance use disorders in a sample of adult substance users. Drug Alcohol Depend.

[2] Johnson K, Pinchuk I, Melgar MIE, Agwogie MO, Salazar Silva F (2022). The global movement towards a public health approach to substance use disorders. Ann Med.

[3] Mulhern JP. Consideration of Social Determinants Risks in Substance Use Disorder Assessment and Treatment Plan Formulation. J Addict Nurs 2022 Jul-Sep 01;33(3):200–2. doi: 10.1097/JAN.0000000000000473. PMID: 36041164.. 10.1097/JAN.0000000000000473 36041164

[4] Olsen Y. What is addiction? History, terminology, and Core Concepts. Med Clin North Am. 2022 Jan;106(1):1–12. PMID: 34823724.. 10.1016/j.mcna.2021.08.001 34823724

[5] McKee SA, McRae-Clark AL (2022). Consideration of sex and gender differences in addiction medication response. Biol Sex Differ.

[6] Shahzad S, Bano N, Begum N, Jones HE (2022). Cultural Adaptation and Validation of the Urdu Version of the cognitive emotion regulation questionnaire (CERQ) in male patients with Substance Use Disorders (SUDs) in Pakistan. Front Psychiatry.

[7] Bøhle K, Otterholt E, Bjørkly S (2021). Protective factors against psychological distress among inpatients in Substance Use treatment: a cross-sectional study. Subst Abuse.

[8] Wangensteen T, Hystad J (2022). Trust and collaboration between patients and staff in SUD treatment: a qualitative study of patients’ reflections on inpatient SUD treatment four years after discharge. Nordisk Alkohol Nark.

[9] Kayaoğlu K, Şahin Altun Ö (2022). The effect of combined cognitive-behavioral psychoeducation and music intervention on stress, self-efficacy, and relapse rates in patients with alcohol and substance use disorders: a randomized controlled trial. Perspect Psychiatr Care.

[10] Bustos-Gamiño M, Mora-Ríos J, Villatoro-Velázquez J, Fleiz-Bautista C, Molina-López A, Medina-Mora ME (2022). Changes in attitudes toward people with Substance Use Disorder: a comparative study of the General Population in Mexico. Int J Environ Res Public Health.

[11] Salani D, Goldin D, Valdes B, McKay M. The Impaired Nurse. Am J Nurs. 2022;Published Ahead of Print. 10.1097/01.NAJ.0000884568.95085.dd. Epub ahead of print. PMID: 36083031.&#8206. 10.1097/01.NAJ.0000884568.95085.dd 36083031

[12] Weber A, Miskle B, Lynch A, Arndt S, Acion L (2022). Services available at United States Addiction Treatment Facilities that offer medications versus behavioral treatment only: a cross-sectional, observational analysis. Subst Abuse Rehabil.

[13] Thal SB, Maunz LA, Quested E, Bright SJ, Myers B, Ntoumanis N. Behavior change techniques in physical activity interventions for adults with substance use disorders: a systematic review. Psychol Addict Behav. 2022 Jun 6. 10.1037/adb0000842. Epub ahead of print. PMID: 35666890.. 10.1037/adb0000842 35666890

[14] Rosenthal A, Ebrahimi C, Wedemeyer F, Romanczuk-Seiferth N, Beck A. The Treatment of Substance Use Disorders: Recent Developments and New Perspectives. Neuropsychobiology. 2022 Jun 20:1–22. doi: 10.1159/000525268. Epub ahead of print. PMID: 35724634. 10.1159/000525268 35724634

[15] Junyue J, Siyu C, Xindong W, Qinge X, Jingchun Z, Liming L, Guohua L (2021). Complementary and alternative medicine for Substance Use Disorders: a scientometric analysis and visualization of its Use between 2001 and 2020. Front Psychiatry.

[16] Moe FD, Moltu C, McKay JR, Nesvåg S, Bjornestad J (2022). Is the relapse concept in studies of substance use disorders a ‘one size fits all’ concept? A systematic review of relapse operationalisations. Drug Alcohol Rev.

[17] Ward J, Hovey A, Brownlee K. Mental health benefits of mounted equine-assisted therapies: a scoping review. Health Soc Care Community. 2022 Jul;10. 10.1111/hsc.13904. Epub ahead of print. PMID: 35811394.. 10.1111/hsc.13904 35811394

[18] Nieforth LO, Craig EA (2021). Patient-centered communication (PCC) in equine assisted Mental Health. Health Commun.

[19] Fridén L, Hultsjö S, Lydell M, Jormfeldt H. Relatives’ experiences of an equine-assisted intervention for people with psychotic disorders. Int J Qual Stud Health Well-being. 2022;17(1):2087276. doi: 10.1080/17482631.2022.2087276. PMID: 35698741; PMCID: PMC9310814. &#8206. 10.1080/17482631.2022.2087276 PMC9310814 35698741

[20] Hemingway A, Carter S, Callaway A, Kavanagh E, Ellis S (2019). An exploration of the mechanism of action of an equine-assisted intervention. Animals.

[21] White-Lewis S. Equine-assisted therapies using horses as healers: A concept analysis. Nurs Open. 2019 Sep 27;7(1):58–67. 10.1002/nop2.377. PMID: 31871691; PMCID: PMC6917924.&#8206. 10.1002/nop2.377 PMC6917924 31871691

[22] Gross JJ, John OP (2003). Individual differences in two emotion regulation processes: implications for affect, relationships, and well-being. J Personal Soc Psychol.

[23] Mauss IB, Levenson RW, McCarter L, Wilhelm FH, Gross JJ (2005). The tie that binds? Coherence among emotion experience, behavior, and physiology. Emotion.

[24] Ochsner K, Gross JJ (2005). The cognitive control of emotion. Trends Cogn Sci.

[25] Schwarzer R, Jerusalem M, Weinman J, Wright S, Johnston M (1995). Generalized self-efficacy scale. Measures in health psychology: a user’s portfolio. Causal and control beliefs.

[26] Schwarzer R, Jerusalem M. (2010). The general self-efficacy scale (GSE). Anxiety, Stress, and Coping. 12. 329–345.

[27] Sorensen MJ. (2006): The Self-Esteem Test. The self- Esteem Institute, pp. 1–5. Steps. Psych Central. Retrieved on May 2, 2017, from https://psychcentral.com/blog/archives/2010/02/25/building-assertivenessin-4-steps/.

[28] Martín-Albo J, Núñez JL, Navarro JG, Grijalvo F (2007). The Rosenberg Self-Esteem Scale: translation and validation in University students. Span J Psychol.

[29] Solmi M, Radua J, Olivola M, Croce E, Soardo L, Salazar de Pablo G, Il Shin J, Kirkbride JB, Jones P, Kim JH, Kim JY, Carvalho AF, Seeman MV, Correll CU, Fusar-Poli P. Age at onset of mental disorders worldwide: large-scale meta-analysis of 192 epidemiological studies. Mol Psychiatry 2022 Jan;27(1):281–95. 10.1038/s41380-021-01161-7. Epub 2021 Jun 2. PMID: 34079068; PMCID: PMC8960395.. 10.1038/s41380-021-01161-7 PMC8960395 34079068

[30] Yüksel BC, Mortan Sevi O. Substance use-related factors and psychosocial characteristics among Turkish adults with early- and late-onset substance use disorder. J Ethn Subst Abuse. 2022 Jun 9:1–22. doi: 10.1080/15332640.2022.2075514. Epub ahead of print. PMID: 35678296.&#8206. 10.1080/15332640.2022.2075514 35678296

[31] Lanza HI, Bello MS, Cho J, Barrington-Trimis JL, McConnell R, Braymiller JL, Krueger EA, Leventhal AM (2021). Tobacco and cannabis poly-substance and poly-product use trajectories across adolescence and young adulthood. Prev Med.

[32] Wakeman SE, McGovern S, Kehoe L, Kane MT, Powell EA, Casey SK, Yacorps GM, Irvin JR, Rodriguez W, Regan S (2022). Predictors of engagement and retention in care at a low-threshold substance use disorder bridge clinic. J Subst Abuse Treat.

[33] McCabe SE, Schulenberg JE, Schepis TS, McCabe VV, Veliz PT (2022). Longitudinal analysis of Substance Use Disorder Symptom Severity at Age 18 years and Substance Use Disorder in Adulthood. JAMA Netw Open.

[34] Witkiewitz K, Pfund RA, Tucker JA (2022). Mechanisms of Behavior Change in Substance Use Disorder with and without formal treatment. Annu Rev Clin Psychol.

[35] Marchand WR, Klinger W, Block K, VerMerris S, Nazarenko E, Curtis H, Newton J, Herrmann TS, Yabko B, Lane J (2022). Mindfulness-based Therapeutic Sailing for Veterans with Psychiatric and Substance Use Disorders. Mil Med.

[36] Aviv TM, Katz YJ, Berant E (2021). The contribution of Therapeutic Horseback Riding to the improvement of executive functions and self-esteem among children with ADHD. J Atten Disord.

